# Supplementary material for: Causal Relationship between Aging and Anorexia Nervosa: A White-Matter-Microstructure-Mediated Mendelian Randomization Analysis
Source: Biomedicines. 2024 Aug 16;12(8):1874. doi: 10.3390/biomedicines12081874 (PMC11351342; doi:10.3390/biomedicines12081874)
Supplement: Supplementary file 1 [file biomedicines-12-01874-s001.zip › Supplementary Figure/Figure S4/Singe SNP plot of MR analysis for TL to ubm-b-1685.pdf]

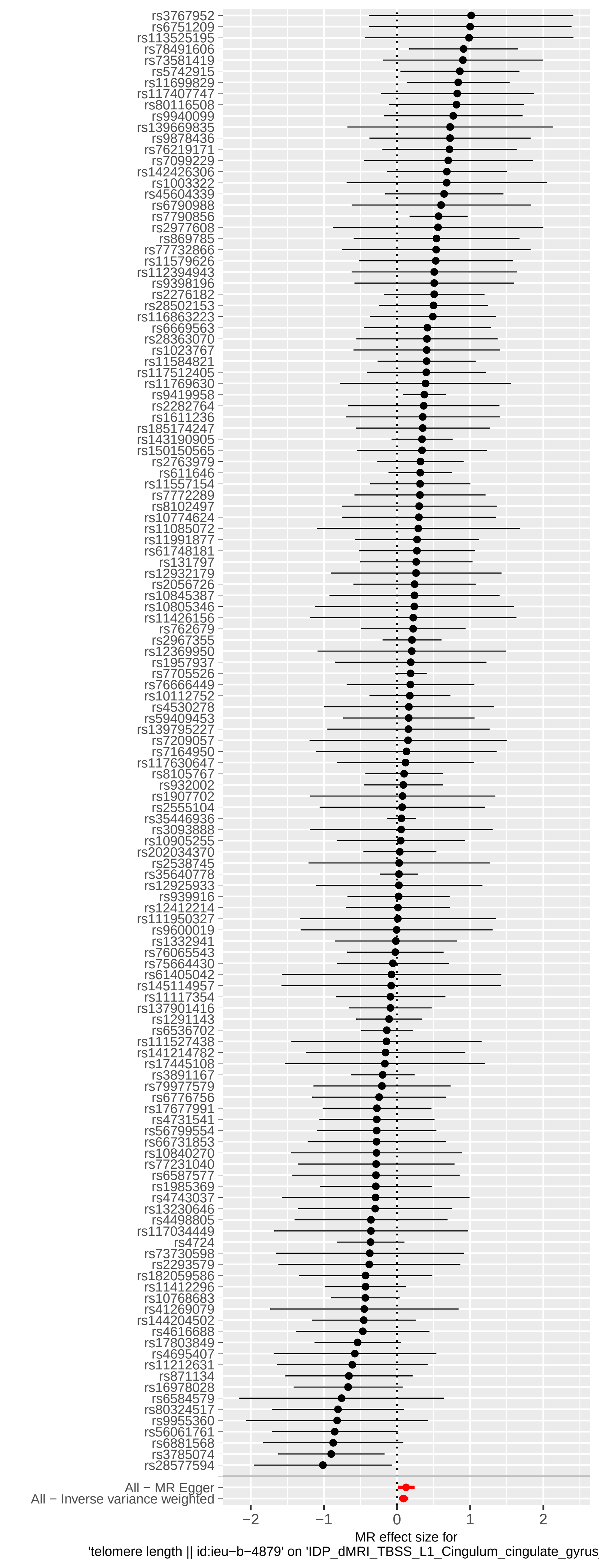
rs3767952
rs6751209
rs113525195
rs78491606
rs73581419
rs5742915
rs11699829
rs117407747
rs80116508
rs9940099
rs139669835
rs9878436
rs76219171
rs7099229
rs142426306
rs1003322
rs45604339
rs6790988
rs7790856
rs2977608
rs869785
rs77732866
rs11579626
rs112394943
rs9398196
rs2276182
rs28502153
rs116863223
rs6669563
rs28363070
rs1023767
rs11584821
rs117512405
rs11769630
rs9419958
rs2282764
rs1611236
rs185174247
rs143190905
rs150150565
rs2763979
rs611646
rs11557154
rs7772289
rs8102497
rs10774624
rs11085072
rs11991877
rs61748181
rs131797
rs12932179
rs2056726
rs10845387
rs10805346
rs11426156
rs762679
rs2967355
rs12369950
rs1957937
rs7705526
rs76666449
rs10112752
rs4530278
rs59409453
rs139795227
rs7209057
rs7164950
rs117630647
rs8105767
rs932002
rs1907702
rs2555104
rs35446936
rs3093888
rs10905255
rs202034370
rs2538745
rs35640778
rs12925933
rs939916
rs12412214
rs111950327
rs9600019
rs1332941
rs76065543
rs75664430
rs61405042
rs145114957
rs11117354
rs137901416
rs1291143
rs6536702
rs111527438
rs141214782
rs17445108
rs3891167
rs79977579
rs6776756
rs17677991
rs4731541
rs56799554
rs66731853
rs10840270
rs77231040
rs6587577
rs1985369
rs4743037
rs13230646
rs4498805
rs117034449
rs4724
rs73730598
rs2293579
rs182059586
rs11412296
rs10768683
rs41269079
rs144204502
rs4616688
rs17803849
rs4695407
rs11212631
rs871134
rs16978028
rs6584579
rs80324517
rs9955360
rs56061761
rs6881568
rs3785074
rs28577594
All − MR Egger
All − Inverse variance weighted
−2
−1
0
1
2
MR effect size for
'telomere length || id:ieu−b−4879' on 'IDP_dMRI_TBSS_L1_Cingulum_cingulate_gyrus
